# Inhibitory Tests as Assessment Tools for Somatic Dysfunctions: Mechanisms and Practical Applications

**DOI:** 10.7759/cureus.7700

**Published:** 2020-04-16

**Authors:** Eduardo Bicalho, Leonardo Vieira, Daniel K Makita, Luis Rivas

**Affiliations:** 1 Osteopathy, Brazilian College of Osteopathy, Sorocaba, BRA; 2 Osteopathy, Brazilian Academy of Fascias, Belo Horizonte, BRA; 3 Osteopathy, Osteopathy School Germany, Hamburg, DEU

**Keywords:** somatic dysfunction, central sensitization, manual therapy, osteopathic manipulative treatment, inhibitory test

## Abstract

The capital element in the field of osteopathy and several other manual therapy methods, is the somatic dysfunction (SD), a functional imbalance that can involve different tissues and mechanisms in its genesis and maintenance. The main challenges found in the clinical scope are to understand the interaction, hierarchy, and relevance of the SD. Several manual tests are available to functionally evaluate the SD, each one with its applicability to analyze the different parameters of the SD. The so-called inhibitory tests are a category of functional manual tests that can be added to the diagnostic context of the SD. It is a particular type of test in which the evaluator applies manual mechanical stimuli to dysfunctional tissues and assesses the biological responses that occur simultaneously with the application of the stimulus. Its use can consider biomechanical and neurological principles in such a way that different conditions can be analyzed. The objective of this article is to review well-established knowledge and recent scientific discoveries about the SD and its local and global repercussions, in an attempt to offer ideas that can be applied to better understand the mechanisms that imply the use of inhibitory tests as complementary clinical diagnostic tools. It will be discussed some of the possible mechanisms involved in the physiology of the inhibitory tests, their practical applications in some distinct conditions, as well as new proposals of utilization based on the sensitization of metameric related structures under a dysfunctional state.

## Introduction and background

The somatic dysfunction (SD) plays a central role in osteopathy and in several other methods in manual therapies. It is a functional disorder considered an obstacle to the inherent self-regulatory capabilities of the human body, characterized by clinical parameters that involve increased tissue “tension/density”, positional “asymmetries”, “restricted” mobility, and “tenderness” (TART) [1]. Several models about the genesis and maintenance of the SDs have been postulated throughout history, conditioning it to neurological reflexes, nociceptive disorders, and recently the neuro-fasciagenic model proposed the role of the modifications in fascial tissue physiology related to the SDs [2-4]. This disorder promotes afferent bombardments from tissue receptors to the central nervous system (CNS), keeping it in a state of alert (facilitated). It supports a neurological loop that generates efferent reflexes to the dysfunctional tissue itself, affecting its neural, biomechanical, fluid, and physiological characteristics. Some authors suggest the close relationship of the SD with the central sensitization phenomenon, in such a way that this state of alertness could keep the receptors of the different tissues that converge their afferent information to the same spinal cord segment involved, with modifications in its activity threshold [1, 5].

Clinical evaluation can reveal several SDs in the same patient, and some of the main challenges are to understand the relevance and hierarchy of these disorders in the individual homeostasis so that the therapeutic approach can be as accurate as possible. Several manual tests that analyze the SD parameters are described in the literature, each presenting its benefits and limitations [6]. They must be used in a combined way, adapting the evaluation process to the individual needs. The inhibitory tests are tools that can be added to the diagnostic context of the SDs, taking into account their biomechanical and neurological aspects [7]. This category of functional test, in which the evaluator generates manual mechanical stimuli in the dysfunctional zones and assesses the possible changes provided by this stimulus, supposedly induces temporary local tissue and/or neurological physiological changes that occur in the manually stimulated tissue [7]. There are different clinical applications of the inhibitory tests, and its objectives are aimed at understanding the dominance and also the interaction of the dysfunctional conditions. This paper will describe some of the possible mechanisms involved in the physiology of inhibitory tests, some of its practical applications suggested by other authors, as well as new proposals based on the convergence of information arising from sensitized segmentally related structures that keep the central nervous system (CNS) in a constant state of alert.

## Review

Somitogenesis: embryology of the segmentation process

Due to the rapid development of the neural tube, a transverse vascular network appears through the dorsal aorta to nourish the high metabolism of ectodermal neural cells. These events are preponderant in the segmentation process [8]. The first somites appear around the 23rd day of the embryo. It is an organization of the paraxial mesoderm around the neural tube. Somitogenesis gives rise to three basic structures: the dermatomes together with the somatopleura, will develop the dermis, layers of fat and superficial fascia; the sclerotomes originate the bones of the body'; and the myotomes which form the deep muscles and fasciae. All somatic structures will carry innervation at the same metameric level [9]. At the same time, viscerogenesis occurs - a process of visceral formation that leads to metameric innervation resulting from the formation of the sympathetic autonomic nervous system and branches of the aortic artery destined to supply the corresponding visceral metabolic demands. Still, at the somite level, there is also afferent information on blood vessels throughout the body carried by sympathetic nerves called spinal vascular afferences [10]. The peripheral nerves themselves are innervated by nociceptive afferences (nervum nervorum), which converge to the posterior horn of the spinal cord through the sympathetic nerves to the corresponding spinal cord levels [11]. Then, a single spinal cord segment or metamer contains innervation of the entire musculoskeletal, visceral, vascular and neural system of the body.

The spinal cord segment and the sensitization mechanisms

A spinal cord segment monitors and integrates different body tissues, promoting an intercommunication network between metameric related structures, such as organs, vessels, nerves, bones, skin, etc [12]. When stress events (injury, inflammation) occur in any of these tissues, inflammatory mediators are released locally, triggering the activity of nociceptors and neurotransmitters that lead afferences to the spinal cord promoting a neurogenic inflammation [13]. Even before reaching the CNS, when these neurotransmitters reach the dorsal root ganglion of the sensory neuron, an antidromic flow occurs back to the peripheral receptor (dorsal root reflex) [14-15]. When potent enough or sustained over time, this nociceptive activity can maintain the alertness of specific neurons in the spinal cord, the wide dynamic range neurons (WDR), making them sensitized for an indefinite period of time [14]. These WDR neurons receive converging inputs from cutaneous, deep somatic and also visceral afferents. This process of neural (central) sensitization can “facilitate” responses from other structures that share converging inputs to the same spinal segment. Shah and Thaker call this clinical condition as spinal segmental sensitization, a hyperactive state of the posterior horn caused by the bombardment of nociceptive impulses from sensitized and/or damaged tissues [16].

Peripheral tissue receptors remain sensitized (peripheral sensitization) due to the dorsal root reflex, lowering their activation thresholds. The consequences of this process are allodynia when a non-nociceptive stimulus causes the perception of pain; and also hyperalgesia, in which a nociceptive stimulus causes an excessive sensation of pain. Hyperalgesia is considered primary when related to tissues that initially cause afferent bombardment and the process of neurogenic inflammation, and secondary when it affects other tissues that converge to the same spinal cord segment (WDR) but are not the primary source of stress [14]. The exaggeration of the perception of pain to a stimulus can last beyond the trigger factor caused by the injury or inflammation of a peripheral tissue affecting the CNS (bottom-up) in such a way that it can be maintained by superior activities of the CNS (top-down) [15].

Some experimental studies have already shown that central sensitization can be primarily promoted by somatic or visceral tissue receptors and that this process can have a direct impact on other tissues that converge inputs to the same affected spinal segment through WDRs that are hyperexcited due to the influence of pain mediating substances such as glutamate and substance P [17-20]. A fracture immobilized for four weeks triggered the activity of afferent C fibers with the release of substance P in the posterior horn of the spinal cord, resulting in chronic glial activation and central sensitization [17]. Immobilization also promoted local tissue changes (allodynia, edema, cutaneous inflammatory mediators), in the afferent sensory neurons (substance P, calcitonin gene related peptide [CGRP]) and in the spinal cord (inflammatory mediators) [18]. The work of Lai and colleagues showed that the skin of the supra-pubic region becomes sensitive to pressure in individuals with urinary tract infection [19]. Sarkar et al. showed that inflammation of the lower esophageal area caused the sensitization of uninjured areas of the same organ by reflex because they are innervated by the same spinal segment [20].

Due to these mechanisms, knowledge about the possible metameric relations between different tissues is of great importance, so that the process of searching for the interactions and degrees of relevance of the SDs can be as accurate as possible. From the potential relationships of a tissue stress/injury and its repercussions in other neurosegmentally related structures described above, it is important to consider that a visceral noxious input can affect the somatic tissues afferences of the same segments, producing symptoms in the locomotor system (viscerosomatic reflex) or in another viscera that converge their neural information (viscerovisceral reflex) [10]. An extreme and interesting example would be the afferences of the colon and rectum that can reach cervical metameric levels (C3-C4), thus being able to produce secondary repercussions in tissues related to these segments (and, therefore, cervical symptoms) due to stress/injury/inflammation in the distal portion of the digestive tract. It is already described in the scientific literature, for example, that when a visceral lesion begins to resolve, the skin is usually the first structure to improve the sensitivity with the rest of the viscera and somatic tissues needing more recovery time [10]. These relationships also occur in the inverse relationship, that is, a lesion in the musculoskeletal system can cause symptoms in other regions of the musculoskeletal system or in the skin (somatosomatic reflex) or also in a viscera (somatovisceral reflex).

The somatic dysfunction - its models and clinical features

The SD is defined as "... the impaired or altered function of related components of the somatic (body framework) system: skeletal, arthrodial and myofascial structures, and their related vascular, lymphatic, and neural elements" [21]. Different hypotheses have been postulated throughout history, initially attributing its genesis and maintenance to the interactions of neurological reflexes. More than a century ago, Burns initiated scientific investigations on the relationship between visceral disorders and somatic reflexes associated with the SDs [22]. Denslow and colleagues evidenced the presence of somatovisceral reflexes in the facilitated areas of the spine related to the palpatory findings of SD [23]. In 1975, Korr proposed the neurological model of the SD, in which he described the “facilitated segment” [2]. For this author, the afferent bombardment originating from proprioceptors caused the “facilitation” of sensory and motor neuronal activity in the segment involved. Subsequently, a model suggested by Van Buskirk in 1990, emphasized the role of nociceptors in the condition of neurogenic inflammation resulting from a stress event (mechanical, chemical, thermal) in the initiation of the process of constant afferences to the spinal segment [3]. In the 21st century, Howell and Willard [13] extended the proposal of the nociceptive model, adding the description of the antidromic action potentials (dorsal root reflex) that occur in the afferent neuron, sustaining inflammatory reactions in peripheral tissue receptors. Fryer proposed the revision of the facilitated segment concept proposed by Korr, due to the scientific expansion in recent decades on the characterization of a phenomenon apparently related to SDs, the central sensitization [1, 2]. For the author, some aspects related to central sensitization (hyperalgesia and allodynia) could explain one of the parameters found in the SD: the increase in tissue sensitivity/tenderness [1]. Recently, the neuro-fasciagenic model proposed the perspective of adding to the neurogenic aspects that have been explored previously, mechanisms related to the role of fascial tissue in the genesis and maintenance of SD in a multidimensional perspective [4]. The model proposes that changes in specific fascia properties should be considered in this context, such as its architecture, contractility, viscoelasticity, fluid content and dynamics, pH, autonomic and somatic neural interactions, metabolic influences, piezoelectricity and epigenetics [4]. The main clinical characteristics of the SDs can be evidenced through manual tests that assess its parameters (TART) [6]. Palpatory sensitivity (allodynia or hyperalgesia) of a dysfunctional tissue is a fundamental condition that can reveal a primary local process, or a secondary neurological metameric reflex possibly related to the central sensitization phenomenon [5].

Diagnostic tools for the SDs and the inhibitory tests

The process of clinical diagnosis of the SDs is performed through manual tests that initially are carried out globally in order to "scan" the body so that areas that need more detailed analysis, from global to local, are found. The segmental tissue evaluation is the final step within the analysis process. Several manual tests that analyze the SD parameters are described to assess each body structure [6]. Among the categories of tests used in clinical practice, the so-called inhibitory tests, consist on the application of manual vector induction stimuli, of a few seconds, that the evaluator applies over an area of SD, to evaluate the immediate distance response over another dysfunctional area, and/or a compensation system, and/or a related function [7]. They can be applied from different perspectives, from the analysis of neurological interactions as well as through local tissue adaptations of the SDs. The temporary inhibition is performed by a manual mechanical pressure on the dysfunctional tissue in order to evaluate immediately any significant changes in functional tests applied previously. Variations in test responses during and/or after the application of the inhibitory stimulus interpret the relevance of the SD in the clinical context. This test category aims to establish the relationship between the identified dysfunctions, differentiating adaptive or compensatory SDs, from the considered primary dysfunctions. It seeks to highlight priority and adaptive dysfunctional patterns according to viscerosomatic, viscerovisceral, somatosomatic and somatovisceral reflexes. Chauffour proposes the use of inhibitory tests on different dysfunctional tissues, and suggests that the physiological mechanisms involved in the inhibitory reflexes provided during the tests need more scientific knowledge, but certainly involve neurological reflexes provided by tissue receptors [24]. Some mechanisms possibly implicated in the physiology of this category of manual tests will be discussed below.

The inhibitory reflex evoked by the touch

Manual contact is often used during the SD evaluation procedures and its perception is a process that occurs through different mechanisms. In addition to the evaluator's haptic perception, touch can also be applied with the intention of temporarily modifying tissue biological activities, so that some repercussions on body systems can be analyzed [25]. The transformation of the mechanical stimuli caused by touch in biological signals occurs through mechanotransduction, one of the several mechanisms by which cells convert mechanical stimuli into electrochemical activity [26]. The mechanical forces are propagated from the extracellular matrix (ECM) to the interior of cells through transmembranous proteins (integrin) and are able to modulate several aspects of cellular function, including its growth, differentiation, migration, gene expression, protein synthesis and apoptosis [26, 27]. Connective tissue penetrates almost every tissue in the body, establishing a direct relationship with the entire system. Cells with contractile capacity are present in this tissue, such as fibroblasts, myofibroblasts and smooth muscle cells, which interact with water, ions, structural proteins. In addition, several types of sensory receptors are present in the ECM, including the multimodals that detect mechanical, thermal, chemical and also nociceptive stimuli [28].

Possibly several mechanisms are involved in inhibitory reflexes provided by the manual soft contact on dysfunctional zones, whether they are triggered at the local tissue level, or even through the activation of different areas of the CNS. Three distinct mechanisms that interact with each other, will be discussed below, and hypothetically are triggered during the application of this category of functional test: 1. the proprioceptive touch; 2. the interoceptive touch; and 3. local and global changes in the fascial tissue.

1. The proprioceptive touch: the mechanical stimulus provided by touch can activate tactile and proprioceptive mechanoreceptors that have low activation thresholds. These receptors inform the CNS by thick-type myelinated fibers of type I and II, or A𝜶 and Aβ, with high conduction speeds (30 to 75 m/s). The role of the activation of these fibers in the process of modulating nociceptive information provided by type C fibers has long been known [29]. The large-caliber fibers have collateral branches that “invade” the posterior horn of the spinal cord and activate interneurons present there (interneurons poll), where the type-C fibers, involved in the sensitization process, also are located. Information from these large-caliber neurons arrives more quickly and intensely in the posterior horn of the spinal cord, changing the synaptic interactions promoted by the nociceptive stimulus, temporarily changing the state of neural sensitization. The “gate control theory”, proposed by Melzack and Wall in 1978, is considered until today, being more and more grounded and with reasoning constructions relevant in the scientific world [30].

2.The interoceptive touch: in addition to the pathways involved by tactile and proprioceptive stimuli, it is plausible to consider the role of pathways that involve the activation of mechanoreceptors innervated by type C unmyelinated fibers, the free nerve endings (FNE) that are spread across the entire body, making up about 80% of the sensory receptors of the fascial tissue [31]. These multimodal receptors have a high adaptive capacity, with different activation thresholds depending on the type of stimulus presenting great sensitization capacity, but with slow conduction speed (~ 1m/s), responding to mechanical, thermal, chemical and also nociceptive stimuli [32]. More intense stimuli with harmful potential for tissues also use these receptors to inform nociception to the upper centers. However, light stimuli, with low activation potential or with non-harmful potential also activate the same receptors, however, changing the information of these neurons in the posterior horn of the spinal cord [33-34]. These physiological characteristics could also be related to the inhibitory reflex triggered by the evaluator's touch in a dysfunctional zone, as this stimulus could alter responses evoked by the FNEs, from a constant harmful/aggressive information over the tissue to an interpretation of “pleasant” non-harmful sensation [35]. The stimulation of these fibers can activate cortical areas such as the left insular, anterior cingulate and left prefrontal cortex, and also promote an inhibition in the amygdala [36]. In addition to other functions, these areas also contribute to the interpretation of interoceptions, emotions, and pain, with the possibility of modifying the initial conditions of the neuromatrix, favoring a different interpretation of pain and central desensitization [33, 36-37]. Therefore, these fibers apparently have a great modulatory capacity of interoceptive information enabling adaptations of the autonomic nervous system, altering the levels of immune cells and cytokines present there [38-39].

3. Local and global changes in fascial tissue: fascia forms a network that surrounds and invests various tissues at different levels, providing form and becoming an element of structural interconnection, nourishing, innervating and allowing the transmission of forces/movement through all body systems, from solid to liquid structures, from macro to micro [40-42]. This connective tissue has viscoelastic properties that adapt its structure according to tension demands, locally and globally. The fascial continuum constantly transmits and receives mechano-metabolic information, which can influence the shape and function of the entire body. The concept of biotensegrity considers this continuity and interdependence of body tissues, as well as the dynamic behavior of cells with the ECM [26, 43]. The SDs promote modifications in several physiological mechanisms of the fascial tissue, such as its architecture, contractility, viscoelasticity, fluid content, and dynamism, pH, piezoletericity, etc [4]. Ingber emphasized that any fascial dysfunction can easily cause repercussions throughout the body, creating stress in any structure involved by the fascia, thus requiring a progressive adaptation of the body at local and global levels [44]. From this perspective, movement is seen in a more complex way, as an adaptive product of the interaction between the systems in order to maintain the lowest possible allostatic load. These concepts support the hypotheses about the mechanics involved in movements, their dissipation of forces and the global connectivity between tissues, which operate concurrently in the control of movements [45, 46]. Therefore, it is plausible to consider that the mechanical stimulus provided by the manual contact in dysfunctional tissue zones can momentarily modify the mechanical stresses of this continuous system that propagates and connects the entire human body, promoting temporary changes in its parameters, such as the densification of the connective tissue, the fluid dynamics and also the trigger threshold of mechanosensitive receptors present in the ECM [47]. In addition, mechanical stimuli produce changes in the ECM capable of inducing local responses in the tissue, such as vasodilation and alteration of its viscosity, enabling the reduction of local sensitization, as well as changes that propagate throughout the fascial continuum [31]. Lunghi suggests that the stimulus applied to the dysfunctional tissue produces changes at the cellular level, promoting changes in gene expression [7]. Tozzi, on the other hand, proposes that when the test is properly applied to a relevant dysfunction, some tissue changes can be perceived, such as the reduction of tension, or even the elimination or temporary reduction of symptoms of the evaluated individual [48].

Practical applications of inhibitory tests as diagnostic tools for the somatic dysfunctions

The inhibitory tests are a category of manual tests that can be applied in the diagnostic scope of the SDs so that therapeutic decisions are made accurately while respecting individual needs. It aims to analyze the relevance of the found dysfunctions, searching for their hierarchy and interactions. Lunghi emphasized that inhibitory tests may also be intended to define the type of approach that will be applied [7]. He highlighted that in conditions of positive inhibitory tests, it suggests a minimalist approach, and in cases of negative inhibitory tests the maximalist approach would supposedly be more appropriate. These tests have a wide range of practical applications, some of which are found in the literature, and others suggested by this paper authors.

**Figure 1:**
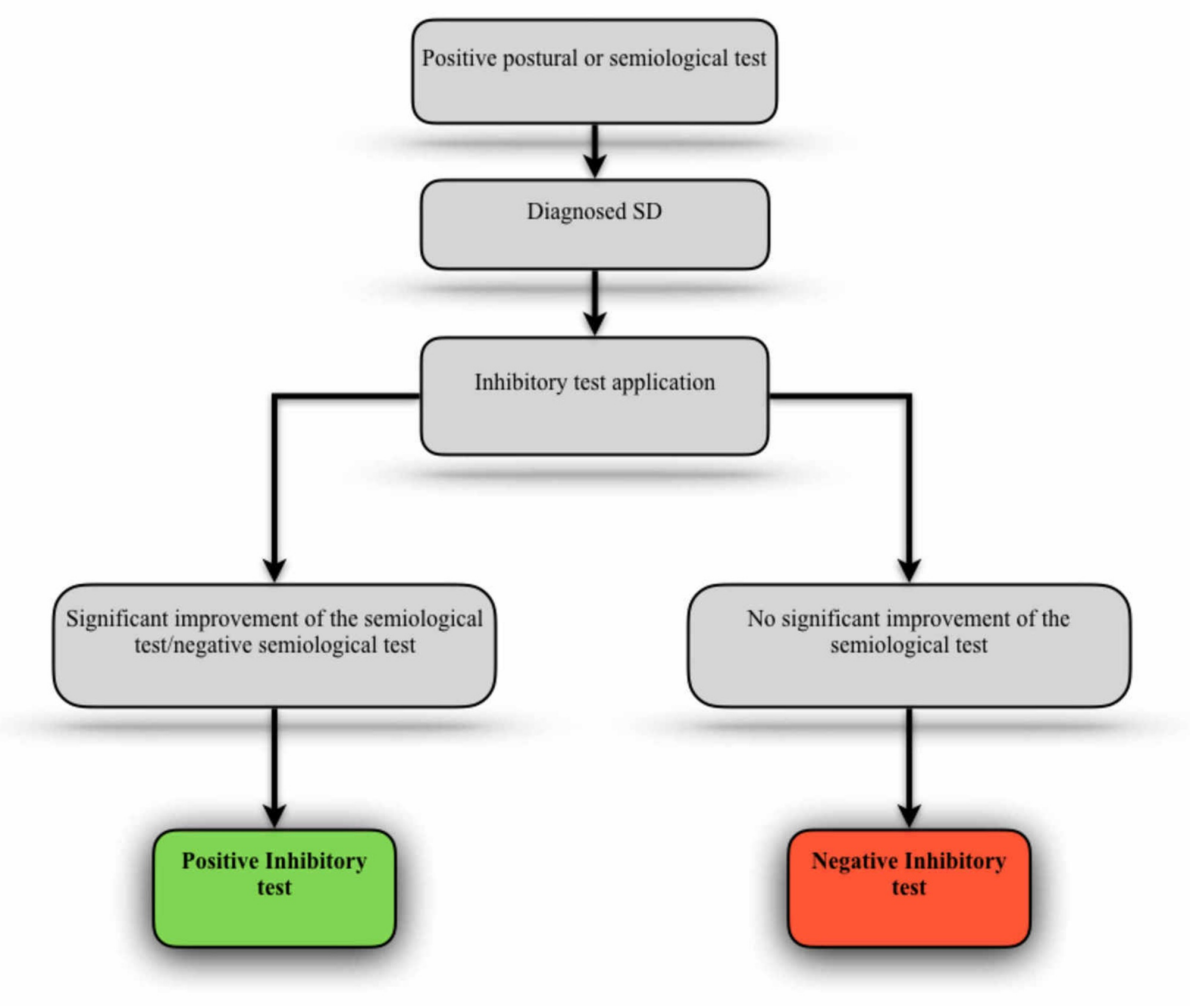
Inhibitory test applied with postural balance or semiological tests SD - somatic dysfunction Adapted from Lunghi and colleagues [25].

In one of these proposals, Lunghi and colleagues propose the application of inhibitory stimuli in dysfunctional tissues so that their relationship with postural balance tests or also with semiological tests (eg Laségue or Soto Hall) can be analyzed [25]. The authors suggest that variations in the test responses, after manual inhibition of the dysfunctional area, can demonstrate the relevance of the dysfunctional structure in the postural context (Figure 1). 

**Figure 2:**
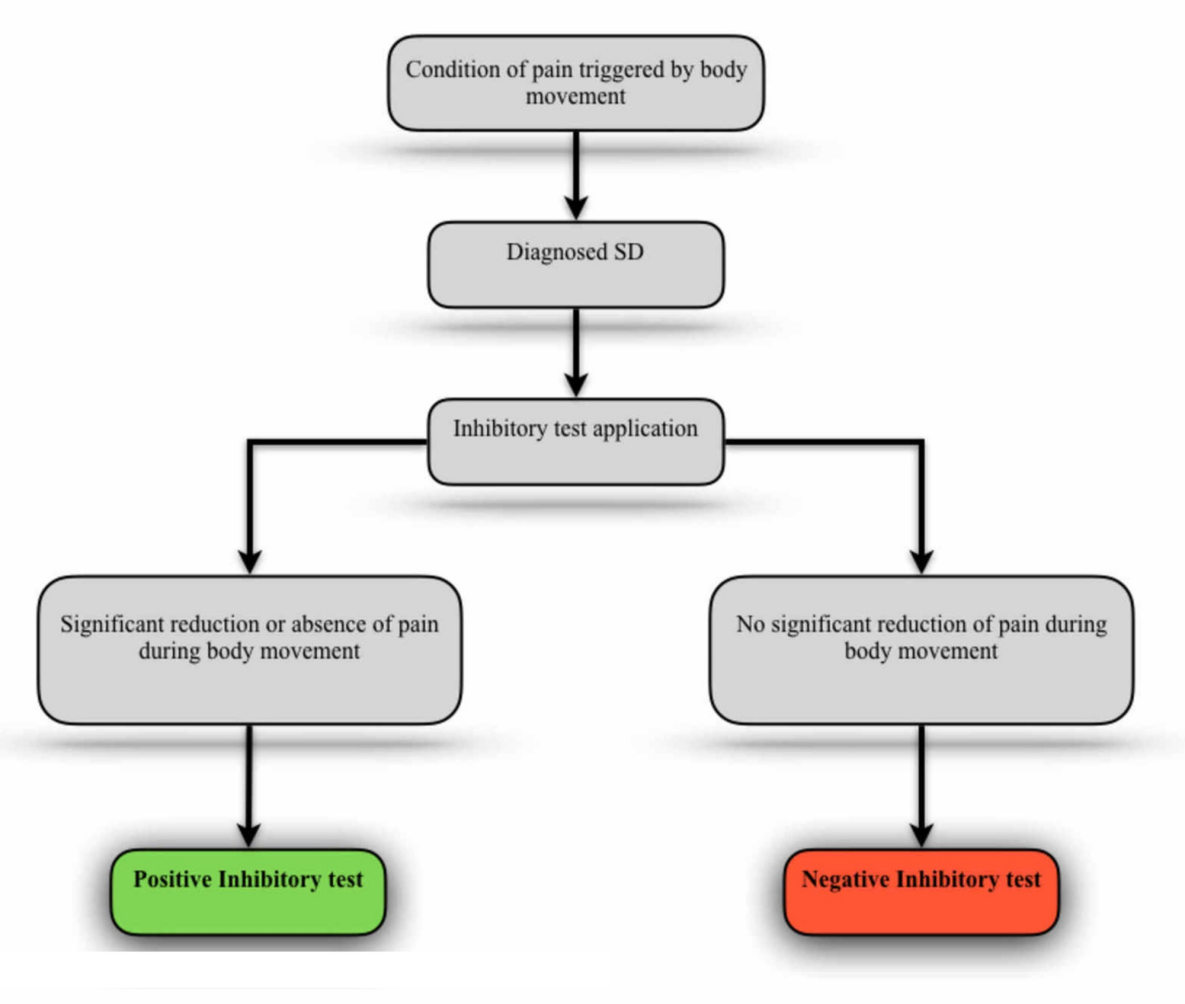
Inhibitory test applied with symptomatic body movement SD - somatic dysfunction

It is also possible to consider the applicability of the test under conditions in which the evaluated individual presents the sensation of pain awakened by any body movement that can be reproduced in the clinical setting. In this case, the inhibitory test would seek to analyze the relationship of SDs previously and properly diagnosed, with the pain condition presented by the individual. To perform this “challenge”, it should identify the body movement that triggers the pain and/or decreased range of motion. Then, as shown in Figure 2, the evaluator should perform the inhibitory stimulus in the dysfunctional structure and simultaneously ask the patient to perform again the compromised body gesture. If the inhibitory reflex is capable of significantly relieve or disappear the sensation of pain and/or significantly amplify the range of motion, the test can be interpreted as positive, showing the relevance of SD in the clinical picture.

**Figure 3:**
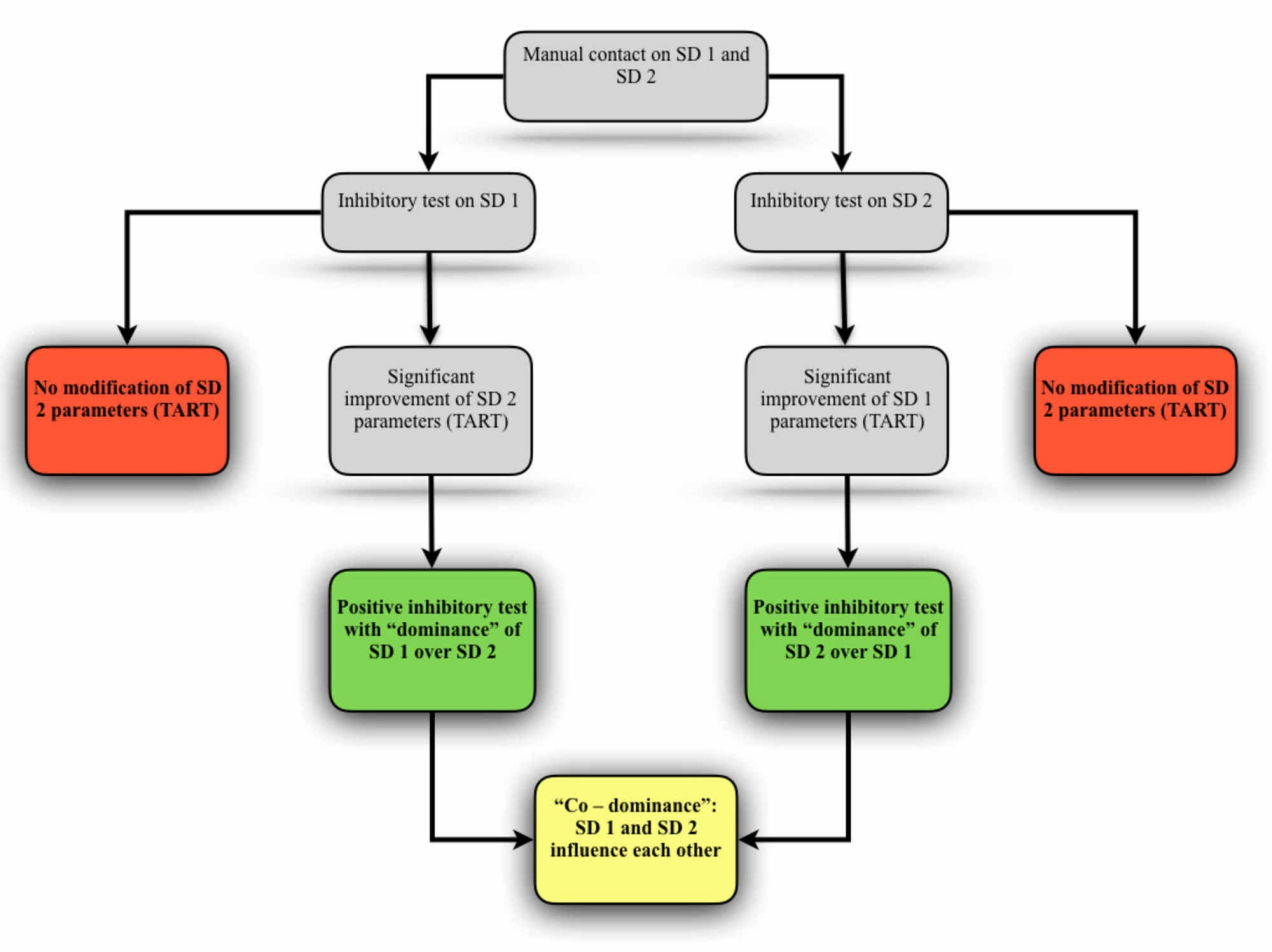
Inhibitory test applied between distinct SDs SD - somatic dysfunction; TART - tension/density, positional asymmetries, restricted mobility, and tenderness Adapted from Chauffour [24] and Fusco [49].

Chauffour and Fusco proposed the use of inhibitory tests in order to establish the dominance and hierarchy of dysfunctional patterns [24, 49]. Its practical application requires prior localization and due to manual contact in two distinct SDs. Applying the inhibition stimulus to one of the SDs, the evaluator must monitor any changes that may occur in the other SD: changes in involuntary rhythms, density, tone or pain sensation (Figure 3). Inhibition temporarily eliminates or reduces the disruptive influences of one SD over another, denominated as a “dominance” condition. The "co-dominance" occurs when both SDs inhibition influences positively each other [50].

**Figure 4:**
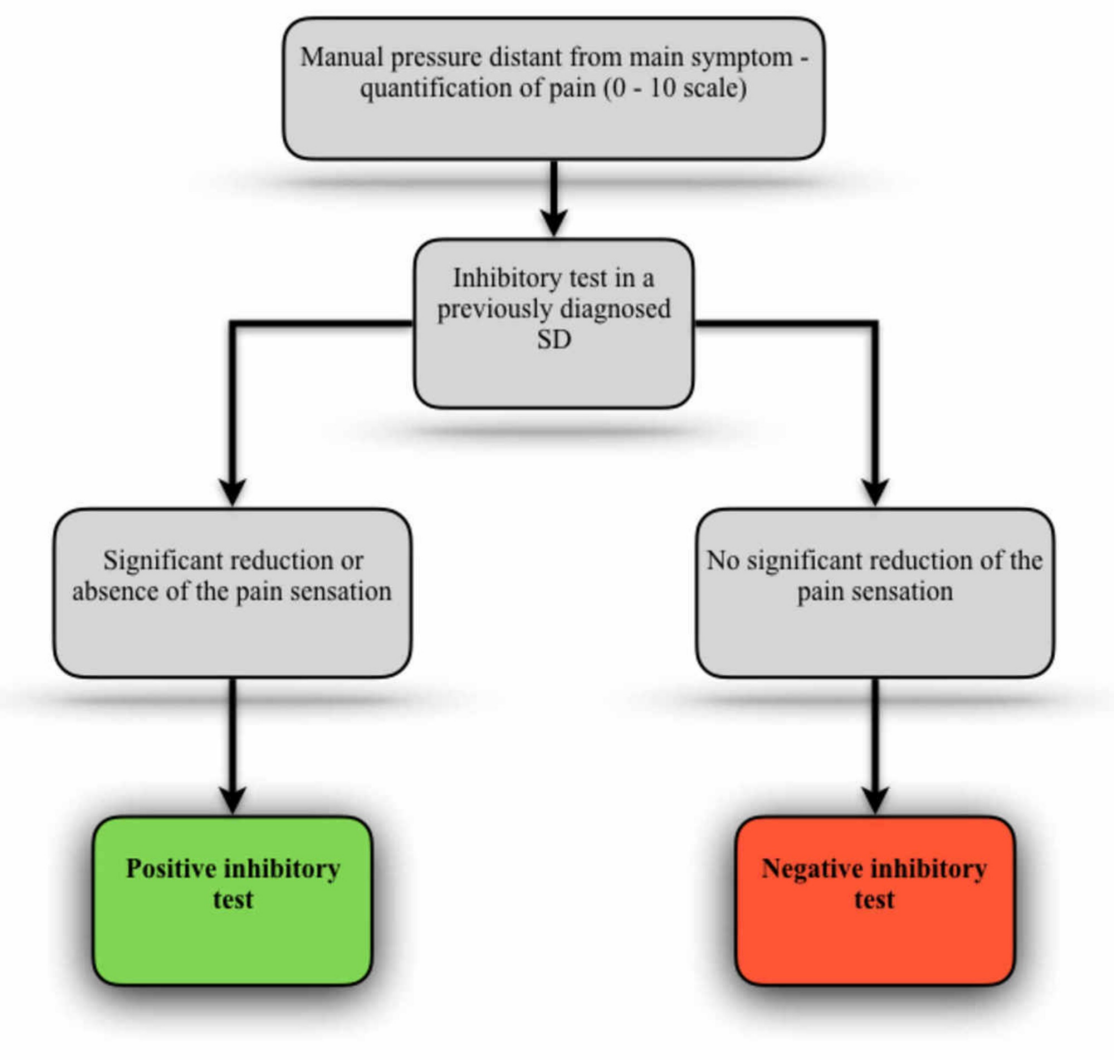
Inhibitory test applied in relation to central sensitization state SD - somatic dysfunction Adapted from Fusco [50].

Following a neurological model, based on the central sensitization phenomenon, Fusco proposes the application of the inhibitory test in order to test the impact of a given SD in the modification of the pain threshold provided by the sensitization condition [50]. To do so, the evaluator should perform a certain manual pressure (4 kg/cm^2^) on a region distant from the patient's main symptom, and ask the patient to quantify the pain on a scale (0 - 10). Then, the evaluator must contact and inhibit one of the previously diagnosed SDs and again apply the pressure in the selected zone previously chosen. If the inhibition of the SD causes a significant reduction or absence of the pain sensation, the test can be interpreted in such a way as to show the relationship of this SD as the general state of central sensitization (Figure 4).

Adding a new possibility, we propose the application of the inhibitory test in another perspective according to a neurological model. This proposal is based on the convergence of aberrant information arising from sensitized metameric related structures. As previously described, the central sensitization phenomenon is followed by modifications in the activation threshold of neurons that converge to the spinal cord segment through WDR neurons, keeping the CNS in a constant state of alert. The main goal of this test is to “challenge” the body and seek the origin of the most relevant afferent bombardment to a facilitated spinal segment, previously and properly diagnosed.

**Figure 5:**
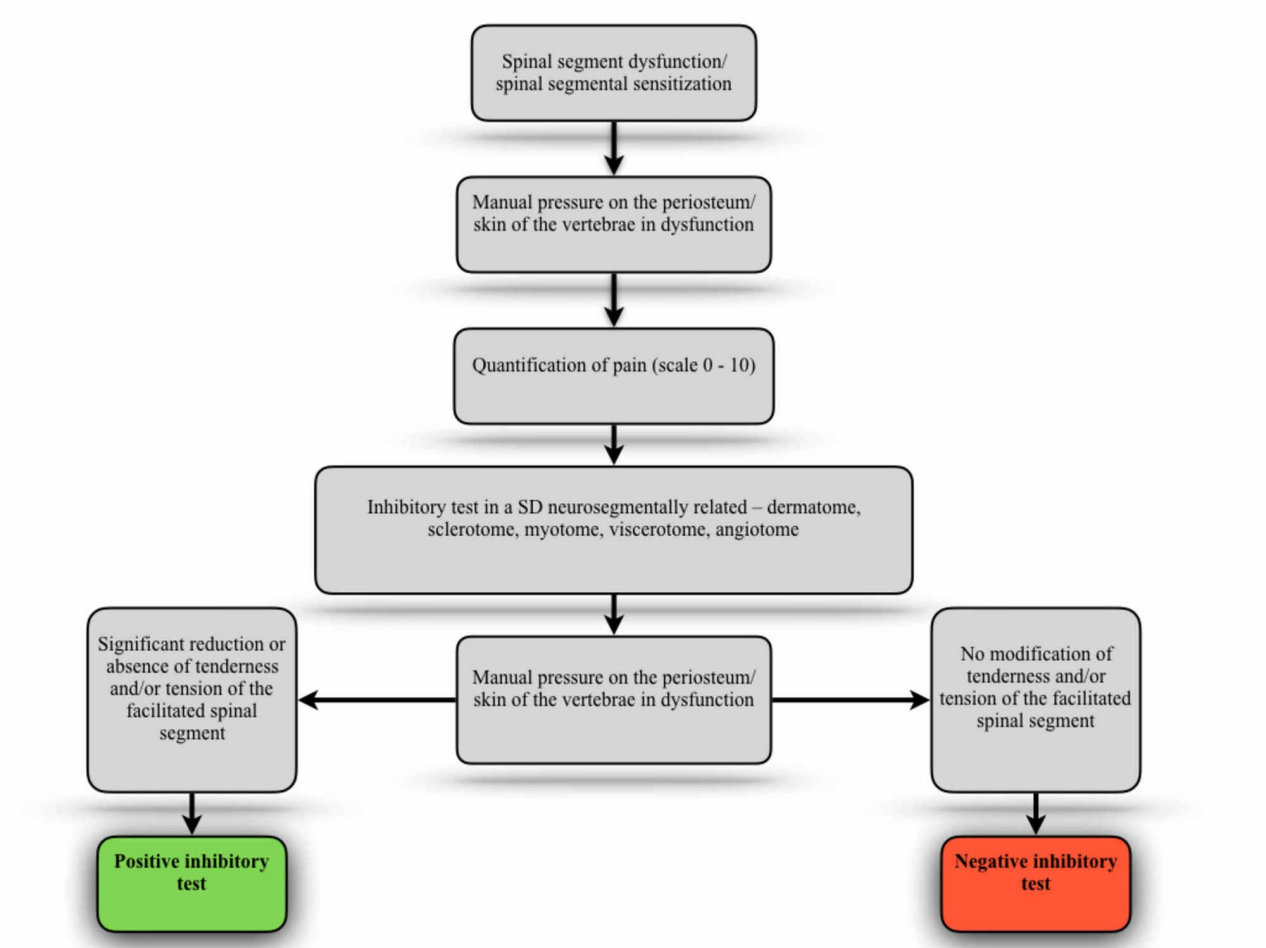
Inhibitory test applied in sensitized metameric related structures SD - somatic dysfunction

It is suggested that the evaluator, after properly reaching a relevant facilitated spinal segment, exerts significant manual pressure on the periosteum (spinous process/lamina) and/or skin of the sensitized spinal segment, and ask the individual being evaluated to quantify the perception of pain (scale 0 - 10) at the moment. Then, the evaluator should apply an inhibitory stimulus in any dysfunctional structure metameric related to the sensitized spinal segment, and that supposedly can be responsible for a primary aberrant afferent activity in the genesis of the disorder presented. The inhibited structure can be the projection of a synovial joint, a visceral fascia, a muscle/tendon, a nerve, a cranial suture, the skin (e.g. scars), a bone, etc. It is recommended that this manual contact applied during the inhibition stimulus does not trigger the perception of pain to the evaluated individual, but only a tactile sensation, so that the CNS might not be bombarded with nociceptive information. Simultaneously with the inhibitory stimulus, the evaluator must again generate the same manual pressure at the vertebral segment initially tested. If the inhibitory stimulus is capable of promoting the disappearance or significant reduction in the perception of pain in the vertebral segment initially tested, it is proposed the interpretation hypothesis that the mechanical stimulus in the tissue primarily responsible for central sensitization could "silence" the activity of the sensitized WDR neurons, including those neurons that conduct afferences from receptors located on the skin and periosteum of the vertebral segment involved in the process. So this test could reveal a possible relevant imbalance in the genesis and maintenance of an alert state presented in the patient's CNS, as it is shown in Figure 5. 

Using the principles of this inhibitory test, the clinical practitioner can also keep in mind the possibility of analyzing whether any SD, found during the evaluation process, has a relevant impact on the patient's CNS. Then, after diagnosing an SD in any structure (somatic, visceral, etc), the evaluator should search for a sensitized spinal segment metameric related. If this interaction is present, the evaluator should seek to quantify the perception of pain in the sensitized spinal segment, then inhibit the peripheral structure in dysfunction and finally analyze the responses of the inhibitory stimulus on the sensitized spinal segment. If the test is positive (significant reduction or absence of pain), it reveals the relevant imbalance condition of the DS on the homeostasis of the assessed individual.

## Conclusions

Based on the characteristics described above, of the somatic dysfunctions and its interactions, its local and global repercussions, and some mechanisms possibly related to the inhibitory reflexes generated by the mechanical stimuli provided by the touch, it is plausible to consider the use of the inhibitory tests within the clinical diagnostic context of somatic dysfunctions as an auxiliary tool. Its distinct ways of application can help the clinician to understand issues related to the relevance, and also the hierarchy of the somatic dysfunctions found so that its approach can be specific and assertive. However, we suggest that scientific studies should be carried out to specifically evaluate the mechanisms evoked by inhibitory tests, its clinical applicability, as well as the advantages and limitations of this manual functional test.
